# The Impact of Exercise on Telomere Length, DNA Methylation and Metabolic Footprints

**DOI:** 10.3390/cells11010153

**Published:** 2022-01-04

**Authors:** Sandra Haupt, Tobias Niedrist, Harald Sourij, Stephan Schwarzinger, Othmar Moser

**Affiliations:** 1 Division of Exercise Physiology and Metabolism, Department of Sport Science, University of Bayreuth, 95440 Bayreuth, Germany; sandra.haupt@uni-bayreuth.de; 2 Clinical Institute of Medical and Chemical Laboratory Diagnostics, Medical University of Graz, 8010 Graz, Austria; tobias.niedrist@medunigraz.at; 3 Interdisciplinary Metabolic Medicine Trials Unit, Division of Endocrinology and Diabetology, Department of Internal Medicine, Medical University of Graz, 8010 Graz, Austria; ha.sourij@medunigraz.at; 4 NBNC—North Bavarian NMR-Centre, University of Bayreuth, 95440 Bayreuth, Germany; s.schwarzinger@uni-bayreuth.de

**Keywords:** telomere length, DNA methylation, metabolomics, exercise, metabolism

## Abstract

Aging as a major risk factor influences the probability of developing cancer, cardiovascular disease and diabetes, amongst others. The underlying mechanisms of disease are still not fully understood, but research suggests that delaying the aging process could ameliorate these pathologies. A key biological process in aging is cellular senescence which is associated with several stressors such as telomere shortening or enhanced DNA methylation. Telomere length as well as DNA methylation levels can be used as biological age predictors which are able to detect excessive acceleration or deceleration of aging. Analytical methods examining aging are often not suitable, expensive, time-consuming or require a high level of technical expertise. Therefore, research focusses on combining analytical methods which have the potential to simultaneously analyse epigenetic, genomic as well as metabolic changes.

## 1. Introduction

Particularly in recent decades, an increase in average lifespan has been recorded globally. Only from 2000 to 2019, global average life expectancy increased from 66.8 years to 73.4 years [1].This phenomenon is multifactorial, including improved management of acute infectious diseases, increased hygiene, improved availability of food, and declining child death [2,3]. While the life expectancy increased by 6.6 years from 2000 until 2019, healthy life expectancy (HALE) increased by only 5.4 years on average [1]. However, chronic noncommunicable diseases such as diabetes mellitus, geriatric disorders or neurodegenerative diseases are increasing, partially due to association of their prevalence with increasing age [4,5,6,7,8].

**Figure 1:**
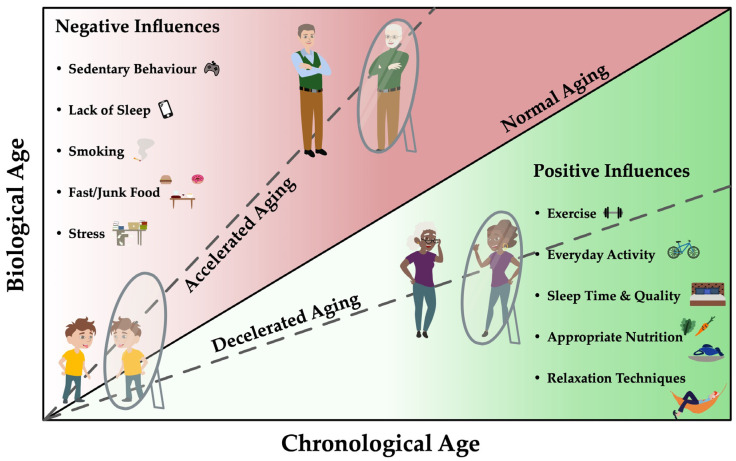
Biological age correlates with the chronological age of a person and depends, among other things, on genetic predisposition, phenotypic changes and epigenetic changes. Nutritional, environmental, psychosocial and other lifestyle (exercise, weight) factors have the potential to both delay and accelerate the aging process and thus modulate risk of diseases. Specific biomarkers could serve as biological age predictors for risk assessment of age-specific diseases and thus influence the aging process positively as well as negatively. This could allow the detection of differences in the risk of age-related diseases for individuals of the same chronological age and identify pathways that have the potential to target these negative epigenetic changes.

For this reason, health care no longer focuses exclusively on extending lifespan; healthy life expectancy has become a focus in health care and research projects [9,10]. In the past years, there is an increasing interest for interventions and reducing the risk of disease in the elderly, which increases with the aging process of the human body. It is assumed that a longer disability-free life can be made possible by impacting cell aging [11]. The aging process of the organism and the accompanying decrease in regenerative capacity, reduced stem cell activity and weakened immune response of the organism [12,13] presumably lead to increased comorbidities burden and limited healthy life expectancy [14] and can be influenced by different (lifestyle) factors, as seen in Figure 1.

The constantly rising prevalence of non-communicable diseases occurs not only due to the aging population but also due to our modern lifestyle [15,16,17]. To characterize the burden of a disease, the concept of disability-adjusted life years (DALYs) has been introduced. DALYs represent the number of years of full health lost due to premature death or morbidity [18]. Dietary risk factors and physical inactivity are mentioned as the most common external causes of increased DALYs [19,20,21]. In most cases, an accumulation of these lifestyle choices leads to disturbances of normal metabolism. Consequences include increased blood pressure, disturbances in lipid metabolism and glucose homeostasis. The prevalence of obesity has almost doubled since the 1980s, and the prevalence of diabetes is around 9% of the world population [22]. The risk for developing type 2 diabetes can be reduced by maintaining a normal body weight and regular physical activity. The World Health Organization (WHO) therefore recommends 150 min of moderate or 75 min of vigorous activity per day [23,24], which, however, is not achieved by the majority of people in the Western world [25]. There is strong evidence that elimination of physical inactivity has not only the potential to prevent or improve metabolic disorders [26,27,28] but also the potential to increase life expectancy as demonstrated in population based analysis [29].

Therefore, this review aimed to summarize current research results on the impact of physical activity on metabolic pathways, genomic and epigenomic factors.

## 2. Materials and Methods

For this narrative review, a non-systematic literature search on PubMed was conducted in October and November 2021 for research studies involving metabolomics, cell proliferation, metabolic pathways and their interaction with physical exercise. Key articles from these areas of research were included.

## 3. Cell Proliferation

### 3.1. Telomeres and Exercise

**Figure 2:**
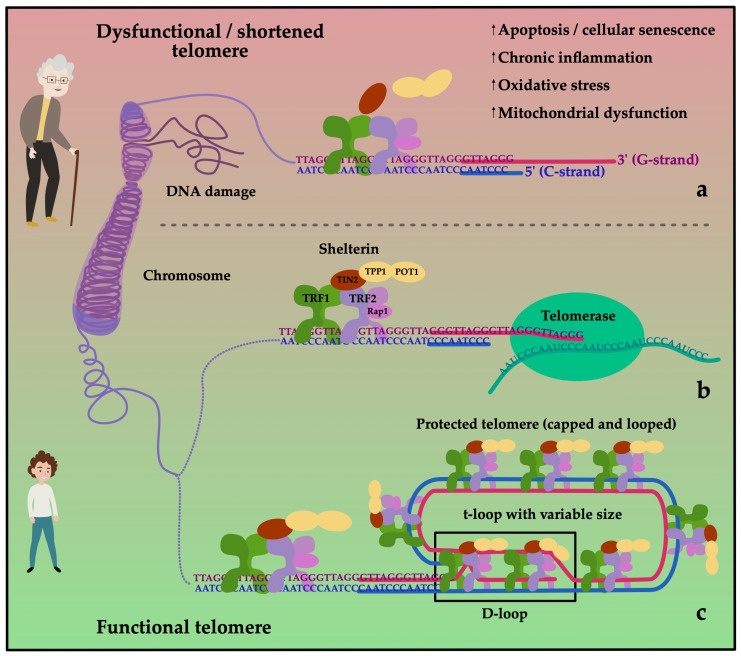
Telomeres act as protective caps at the end of chromosomes. They consist of a sequence of 6 nucleotides (G-strand: 5′-TTAGGG-3′; C-strand: 3′-AATCCC-5′) repeated several thousand times. Telomeres loses length with each cell division. To prevent excessive shortening, they are protected by shelterin. Shelterin is a protein complex consisting of six subunits: telomere repeat binding factor 1 (TRF1), telomere repeat binding factor 2 (TRF2), protection of telomere 1 (POT1), repressor/activator protein 1 (RAP1), TRF1- and TRF2-interacting nuclear protein 2 (TIN2) and tripeptidyl peptidase 1 (TPP1). The 3’ end of the G-rich strand extends over the end of the C-rich strand (5’ end) of the telomere. Given sufficient length of the telomere, this forms the t-loop, which overlaps with the double-stranded 5’ end, building the D-loop protecting the telomeres (**c**). During replication, the G- and C-strands are open. Telomerase can bind to the G-strand to add telomeric repeats preventing the cell from damage (**b**). Loss of function and degradation of the shelterin complex leading to DNA damage occur more frequently during aging and cause the cell to stop dividing (**a**).

Cellular senescence is inevitably connected to telomere biology. Telomeres are repetitive deoxyribonucleic acid (DNA) sequences capping the ends of chromosomes and protecting them from degradation. Every cellular division leads to a shortening of telomeres. When a critical telomere length (TL) is undercut, the cell cannot replicate anymore, and it has become “senescent”. The enzyme telomerase counters telomere attrition by reading telomeric sequences to the shortened strand after each division. Thereby, a high telomerase activity (TA) can delay reaching the state of cellular senescence [30]. The telomeres themselves are protected by protein complexes called “shelterins”, with the most notable proteins being TRF1 (telomere repeat binding factor 1) and TRF2 as shown in Figure 2. These shelterins regulate TA on the telomere and counteract telomerase to avoid overly excessive telomere elongation [31].

Although TL can be measured in any cell, whole blood is by far the preferred material to work with. Besides the practical aspect of easy sampling, it is widely presumed that leukocyte telomere length (LTL) accurately reflects the overall senescence state of an individual. It was recently shown that a positive correlation between LTL and the TLs of most solid tissues exist [32]. From an analytical point of view, the determination of TL is possible through a number of different approaches. However, these methods and their results are not comparable between each other. While some protocols measure the lengths of single telomeres, others determine an average TL [33]. Results are either expressed as absolute or relative TL. The most commonly used method for the determination of LTL is based on quantitative polymerase chain reaction (qPCR). It quantifies the ratio of telomere length to a single copy gene (T/S) which is proportional to the average TL in a cell. This method is quite easy and feasible but lacks standardization, the variations of results may be huge between different laboratories [33]. In contrast to the qPCR, other methods such as terminal restriction fragment analysis (TRF) or protocols based on quantitative fluorescence in-situ hybridization (Q-FISH) may be better standardized and therefore more comparable between different laboratories; but they are also costly and demand highly trained analytical technicians along with expensive laboratory equipment. The current pinnacle of telomere length analysis are measurements which quantify the length of a cell’s shortest telomeres. Such analysis are conducted by few laboratories only and exhibit the most exclusive research character of all TL measuring approaches [34].

Compared to the variety of TL measurement methods, the determination of TA is more challenging. First, TA cannot be detected in many cell types. With the exception of stem cells and tissues which proliferate on a constant high-speed rate, most somatic cells have no measurable or low TA. In human blood however, lymphocytes show high and measurable levels of TA (LTA). Method-wise, the determination of TA is mostly performed using modified versions of the so-called “telomere repeat amplification protocol” (TRAP). In analogy to many TL measuring methods, the results for TA measured with TRAP aren’t directly comparable among different laboratories and therefore not applicable in human routine diagnostics [35]. The real challenge of LTA determination lies within the preanalytical sample preparation: the analysis is set to occur in peripheral blood mononuclear cells (PBMCs) which have to be separated and isolated out of freshly drawn blood samples [36,37]. Specific sampling tubes and agents are required for this purpose and the samples have to be worked on quickly after being drawn. This simply explains why LTA isn’t measured as often as LTL in research projects. While LTL can also be determined out of whole blood samples which have been stored for long time periods, LTA analysis cannot be performed in a retrospective or secondary manner if it wasn’t included in the research planning from the start.

The analytical investigation of shelterins follows two different basic principles: either the expression of a shelterin protein or the protein itself as the expression product is measured. The quantification of the latter can be performed via an enzyme-linked immunosorbent assay (ELISA) in simple serum samples and is by far the easiest laboratory method to conduct compared to the others mentioned in this review. Conversely, gene expression analysis is a much more sophisticated approach and, again, requires serious preanalytical planning and specific laboratory equipment.

Several independent investigations have drawn connections between LTL and physical exercise or overall fitness. A few of these investigations were conducted in well-characterized pathological cohorts, most of them involved elder individuals. A substudy of 582 participants of the Cardiovascular Health Study revealed a positive association between the walking distance and LTL [38]. Furthermore, a longitudinal increase of leisure time physical activity led to a trend towards slower telomere attrition in the same study. In a similar age group of 117 women, who were recruited from the participant pool of the Yonsei Aging Study, LTL was shown to positively correlate with the participant’s walking speed [39]. In a clinical study of elderly patients with knee osteoarthritis, a reduction of gait speed was also observed in those with shorter telomeres [40]. The number of daily steps was associated with higher LTL results in 2312 individuals of the Strong Heart Family Study [41]. Multiple studies on telomere length and physical fitness were conducted within the collective of participants of the National Health and Nutrition Examination Survey (NHANES). The determination of LTL in 2410 individuals aged between 50 and 85 years showed an increased knee extensor strength in the fourth LTL quartile [42]. Another investigation of 1764 adult NHANES participants aged below 50 years showed, that long telomeres are more likely to be present in persons with a high degree of cardiorespiratory fitness objectified by a treadmill test [43]. Similar associations were observed in an even younger population involving more than 4950 participants of the Northern Finland Birth Cohort 1966. Analysis of blood samples obtained at the 31-year follow-up visit revealed a longer LTL in individuals who also displayed high-level aerobic fitness (measured by a 4-min step test), good trunk muscle endurance (Biering-Sorensen test) or handgrip strength [44]. LTL may also play a role for the performance in high-profile sports [45]. Blood analysis of 22 male master track and field athletes showed a surprisingly significant correlation between an athlete’s LTL and his personal relative performance level. The latter was expressed as percentage an athlete’s best performance had in relation to the respective world record.

While the studies mentioned thus far tried to associate LTL with objective measures of physical activity in defined human cohorts, others relied on targeted interviews or questionnaires to characterize an individuals’ lifestyle or sports behaviour. In a NHANES substudy, movement-based behaviour was monitored in 6503 adult participants at four points in time during an interval of four years [46]. The more individuals engaged in physical activities, the less likely it was to find them in the lowest LTL tertile. Sports anamnesis conducted with 815 participants (mean age over 61 years) in the Berlin Age Study II revealed, that active leisure time physical activity or regular sports for at least 10 years prior to the study was clearly associated with higher LTL values [47]. Leisure physical activity was also observed being accompanied by greater LTL in a large population of 7813 women aged over 43 in the Nurses’ Health study [48]. A similar study of a comparable population of 239 postmenopausal women came also to the conclusion, that healthy behaviour such as physical activity reduced TA in a one-year interval [49].

Long-term endurance performance and probability of survival are strongly correlated with TL, but due to many study designs, the question arises whether longer TL is the cause or consequence of a longer and more active life. There is evidence that both maximum oxygen uptake (VO_2max_) and long-term endurance capacity show a positive correlation and most likely have a protective effect on muscle TL in older groups of individuals, resulting in increased longevity [50]. In some cases, the biological age of these groups differs significantly from their actual chronological age. However, the TL shows also strong considerable interindividual variability, which clearly complicates the interpretation of the results [51]. However, the unclear situation regarding the cause-and-effect mechanisms of the examined cohorts and the interindividual differences between studied groups makes it necessary for interpreting the results on individual basis and for including further factors besides the TL in the considerations. For this reason, research also focuses on other aspects in order to obtain a more comprehensive understanding and more conclusive results. In recent years, for example, the degree of DNA methylation, which is directly related to TL, has been also investigated [52,53].

### 3.2. DNA Methylation and Exercise

Cell proliferation in eukaryotic systems is strongly related to epigenetic changes in DNA, which control, among other things, the transcription or repression of genes. This is not a uniform change over the entire genome, but depending on different external factors, e.g., diseases in which only partial areas of the DNA are affected [54]. These epigenetic changes are induced by methylation of genome, which occurs mainly at the fifth carbon atom of the cytosine (5mC) [55]. Cytosines are usually preceded by a guanine or CpG dinucleotide—a combination of two nucleotides containing the base guanine and cytosine. The degree of methylation of human tissue is low, which is attributed to the potential mutagenic effect of 5mC, caused by its large potential to mutate to tyrosine and thus lead to CpG deficiency [56]. However, CpG is essential in the control of genes. The DNA has sections with a particularly high density of CpG dinucleotides, which are called CpG islands. A large part of the gene promoters are located in these partially methylated sections of the DNA sequences [57]. Mutations in these areas thus appear to have an enormous influence on the natural mechanisms of the regulatory action of the genes and therefore on cell proliferation. Fluctuations in DNA methylation and demethylation are natural processes associated with the normal aging of the human body [58,59]. Excessive alterations in this steady-state are, however, closely linked to the development of lifestyle diseases such as cancer [60,61,62], type 2 diabetes [63,64], or autoimmune diseases such as type 1 diabetes [65], drug-induced lupus-like diseases [66], multiple sclerosis [67] or Crohn’s disease [68]. New therapeutic approaches attempt to restore the original methylation-demethylation balance [69,70,71]. Research by Horvath and Hannum et al. identified the measurement of DNA methylation age (DNAmAge) as a suitable method to determining biological age. Using their quantitative model, Hannum et al. showed that the aging rate of tissue is influenced by genetic variants and that tumour tissue exhibits advanced aging rates [72]. This is confirmed by the study of Horvath, which detected a significant age acceleration of an average of 36 years of the examined samples of cancer tissue [73]. Whether disease-promoting epigenetic changes can be influenced by lifestyle measures, such as appropriate nutrition and regular physical activity, or environmental influences and psychosocial factors, has been studied increasingly in recent years in the literature [74,75,76,77]. For example, Anderson et al. showed in their review that the intake of sufficient amounts of folate, choline, betaine of different B vitamins and methionine has a direct influence on the global methylation level and also the methylation of disease-relevant promoters [75]. Although the review focused on animal studies, these approaches demonstrate the importance of understanding our diet at the molecular level. However, data on the association of exercise with DNA methylation remain inconclusive. As already described by Voisin et al. this could be due to the frequently chosen observational study designs [76]. These are often used due to cost reasons and the enormous time required for interventional studies [78,79,80]. Only a weak correlation was found between the degree of methylation and physical activity, independent of the health status of the subjects studied. Reasons for this could be recall bias in the assessment of physical activity, which can occur especially when longer time periods in the past were assessed [81]. Moderate correlations were also found in studies that measured physical activity using accelerometers or pedometers [82,83]. Since these studies investigated the degree of methylation in blood, it also cannot be said with certainty whether there is a correlation between physical activity and DNA methylation in muscle or adipose tissue. In contrast to the observational studies, Voisin et al. found a strong correlation between methylation levels and physical activity in interventional studies [76]. Genes that showed a significant change after exercise included peroxisome proliferator-activated receptor-gamma coactivator 1α (PGC-1α) involved in the regulation of metabolic activity, myocyte enhancer factor 2A (MEF2A) involved in muscle growth, runt-related transcription factor 1 (RUNX1) affecting blood formation, and apoptosis-associated speck-like protein (ASC gene) affecting inflammation. However, there were also differences between the studies considered. Voisin et al. explained the inconsistency between the different results with various factors [76]. There is a problem of selection bias and memory errors in observational studies, and conversely, the selection of suitable test material, which may often not be representative for the investigated research question. Frequently, blood is used as the test material in such studies, since sampling for the analysis of DNA methylation of skeletal muscle must be performed invasively via muscle biopsies—blood sampling is much less time-consuming, less invasive and less expensive. Furthermore, different genes can be examined with the used methylation assays. Thus, there is a possibility that research with low or no correlations did not examine genes specific to physical activity because they were limited to a single tissue examined or the choice of genes for analysis was not representative [76]. A major problem in the examination of genome-wide or epigenome-wide changes is also the fact that many of the studies were either performed with a sufficiently large sample size but insufficient genome coverage, or with sufficient genome coverage but too small sample sizes to detect responses of tissues on interventions when they are only small [84].

Most articles in the literature distinguished between acute and chronic response to exercise. For Example, Barrés et al. studied a single bout of exercise on a cycle ergometer in 14 male and female participants [85]. They took 3 samples—the first before breakfast as resting muscle biopsy. Four hours after consumption of a high-carbohydrate meal the test took place until a defined overall energy expenditure of 1670 kJ was reached. Sample two and three were taken immediately after and 3 h after the test. They showed that after a single bout of exercise, in addition to a local hypomethylation of promoters for metabolic genes (e.g., PGC-1α), a global change in the methylation state of the DNA could be detected [85]. Another study, focused on a single bout of exercise, the methylation level of the nucleotide-260 (nt-260) of PGC-1α was examined [86]. In type 2 diabetics, a hypermethylation of this specific nucleotide of the transcription activator PGC-1α can be observed. Hypermethylation is associated with a reduction in mitochondrial DNA content, which is consistent with the reduced mitochondrial content in people with type 2 diabetes [87]. For the study 11 healthy men had to complete a single bout of exercise on a bicycle, in which they expended an amount of 650 kcal [86]. Bajpeyi et al. found an interindividual heterogeneity in the methylation level of nt-260, which they attributed not exclusively to the genetic variance of the test subjects, but which reflected individual responses to physical training. By dividing the subjects into responders and non-responders on the basis of the different acute training-induced epigenetic changes, they attempted to explain the differences in the trainability of the subjects [86]. An epigenome-wide association study (EWAS) was performed by Seaborne et al. in 8 men, who detected significant changes at over 17,000 CpG sites after a 7-week strength training intervention [88]. They identified over 9000 sites that were hypomethylated and over 8000 sites that were hypermethylated compared to the initial measurement. After a subsequent training break of seven weeks, another 7-week strength training program was completed. There was no change in the number of hypermethylated CpG sites, while the proportion of hypomethylated DNA increased to over 18,000. The largest increase in gene expression after resistance exercise was detected after the reloading phase. They concluded that the muscle cells have a certain epigenetic memory of these specific gene methylation signatures from earlier hypertrophy. Another study by Lindholm et al. incorporated a control group in their observations [89]. Twenty-three non-regularly trained study participants underwent single-legged knee-extension exercise training over a period of 3 months (4 sessions per week, 45 min each). The leg to be trained was randomly selected, while the non-trained leg served as an intraindividual control leg. This enabled them to eliminate external influences such as changes in diet or undetectable environmental factors which could lead to outcome bias. It was shown that the 3-month training period resulted in changes of 4919 methylation sites. Over 800 of these sites showed a change in methylation level of at least 5%. As a result, the transcription of 4076 expressed genes was increased. A large number of other studies are summarized and discussed in the literature. However, they are also limited by their small sample sizes, which makes it difficult to provide valid results [76,90,91]. In addition, the differences in methods used for analysis of methylation levels, the different tissue samples used and the different DNA loci examined are discussed as possible sources of variability in the findings and conclusions [92]. Another source of error for misinterpretation are interindividual differences in the methylation level, which could be masked by a too small sample size. For example there is a disagreement in the literature about the correlation of the methylation level with gender [93,94,95]. El-Maarri et al. in contrast showed in their study with 96 age-matched healthy women and men a clear difference between male and female in the overall methylation level [92].

The presented study results demonstrate the difficulties of the analysis of the sample substance and the interpretation of the resulting outcomes, which do not always allow clear conclusions to be drawn. Here, as seen in research of TL, newer research approaches are moving towards combining several analytical methods in order to enable deeper insights through their synergies.

## 4. Metabolomics

The constantly increasing interest in physiological processes has led to the development of promising analytical methods. Whereas until a few years ago it was only possible to detect single metabolites, today the individual metabolic response can be determined in dependence of genetic and external factors (nutrition, lifestyle, culture, drugs). In this context, a classification is made between proteomics, transcriptomics, genomics and metabolomics, according to the compound under consideration. Omics technologies examine the entirety of all simultaneously occurring processes in the body, which makes it possible to detect the ever-changing multiparametric responses of the organism [96,97]. Metabolomics is probably the most important area from a biochemical point of view. The aim is to map the overall metabolic pathways of individual organisms to specific interventions. These reflect gene, proteome and metabolite activity in the human body [98]. To maintain metabolic homeostasis during exercise, specific tissues must respond to exercise-induced energy demands [99]. Metabolomics makes it possible to obtain a snapshot of the processes occurring in the entire organism. A major problem, however, is the strong inter-individual variation in metabolism—the specific metabotype [100,101,102]. Nevertheless, modern analytical methods are able to distinguish between the metabolic responses to different types of physical exercise such as cardio or resistance training. The measurement of selected metabolites, transcription factors, proteins or lipids shows the different metabolic responses of different phenotypes on a molecular level [103]. Based on these findings, it should be possible to make recommendations for training and nutrition strategies in the context of the health and performance outcomes of physical exercise. Most interesting for the investigations is the individual metabolic responsiveness to different exercise interventions, in order to adapt for example the macronutrient timing to the physical activity and time-dependent patterns in metabolic pathways within the muscle and the systemic energy homeostasis [104], or influence health status in positive way, especially in the prevention and treatment of civilization diseases.

### 4.1. Metabolomics and Noncommunicable Diseases

The interplay between metabolic homeostasis and the resulting adequate immune response plays a crucial role in maintaining a healthy organism [105]. Both are closely linked and disturbances in either system are often associated with the development of diseases or the aging process [106,107,108]. Since life expectancy in humans has increased over the centuries understanding the pathophysiology of diseases and aging is necessary for enhancing disability-free years in the elderly.

The human metabolome is age dependent and therefore specific information about age-related changes in metabolism can be detected in metabolomic profiles [109,110]. Characteristic metabolite profiles have already been identified for various diseases, which can be used not only for disease diagnosis [111,112,113] but also for monitoring the progress of therapy [114,115].

It has long been known that regular aerobic exercise or a combination of aerobic and anaerobic exercise has the potential to improve cardiovascular health and thus minimize the risk of disease [116,117] and is therefore recommended as a supportive therapy for diseases such as cancer [118], cardiovascular disease CVD [119] and type 2 [120] and type 1 diabetes [121,122]. However, the molecular mechanisms behind the positive physiological adaptations resulting from endurance training are still not fully understood. Metabolomic studies could help remedy this and give us a better understanding of disease pathogenesis and its control. For example, Burke et al. were able to detect over 200 different metabolites, which showed altered blood concentrations after an intensive training session. Among them was niacineamide, which plays a crucial role in the metabolism of nicotinamide adenine nucleotide (NAD+/NADH). It is discussed whether an increase in NAD+ concentration can mimic the positive lifespan extending effects of caloric restriction [123]. The possibility of using exercise as a pharmacological-like approach is referred to as “gymnomimetics” [124].

### 4.2. Metabolomics and Exercise—Endurance vs. Strength vs. Relaxation Exercise

Literature contains a large number of metabolomic studies during and after exercise—numerous reviews are already available [125,126,127]. Nevertheless, in the following, exemplary studies will be considered, which show the potential of metabolomic investigations for the comparison of the metabolic response to different types of exercise. The majority of investigations in literature are limited to aerobic sports, such as running, swimming and cycling. Only a small proportion of research focused on resistance training. Compensatory sports such as yoga, which are often used for regeneration focused on different relaxation techniques, were insufficiently described in the literature. In a large pooled-analysis assessing different cohorts including patients and healthy individuals after full exhaustion, exercise-induced increase in glycerol was correlated with fitness levels in the patient group completing the acute exercise testing and in marathon runners. In contrast, in the patients with myocardial ischemia glycerol was decreased. The metabolites found to be altered by exercise directly affected a transcription factor whose suppression has been linked to obesity and diabetes in animal studies. It was concluded that these metabolites could have the potential to be therapeutic for CVD and other diseases [128]. An increase in lipid metabolism was also detected in 14 highly trained athletes (9 men, 4 women) during three identical submaximal tests of 3 min each at 30%, 40%, 50%, 60%, 70%, and 80% of VO_2max_ on a cycle ergometer [129]. Tests were performed in a cross-over design consisting of a control test (3 h after a standardized breakfast, CON), a fasting test (12 h after a standardized evening meal, FAST), and a postexercise test (after a standardized breakfast, endurance training, and 2 h of fasting recovery in between, EXER). During EXER, maximal fat oxidation rate, blood insulin, free fatty acid, and cortisol levels were higher compared to CON and FAST, suggesting that exercise significantly increases whole-body fat oxidation and is not only due to overnight fasting. The results found are in line with previous research [130,131]. A study of 10 men performing 4 sets of 10 repetitions at 70% of a repetition maximum demonstrated that resistance exercise (RE) also produces a specific pattern of metabolites characterized by increased 2-hydroxybutyrate, alanine, lactate, pyruvate, and succinate concentrations [132]. An increase in pyruvate and lactate in the blood was detected immediately after the single exercise session. Both compounds are formed during anaerobic glycolysis as a result of high-intensity exercise. In the trial metabolites related to energy metabolism could be found about 5 min after RE, while others, such metabolites related to anabolic processes and recovery, decreased about 1 h after exercise.

How the altered metabolic processes during regular physical activity affect the physical performance of the individuals in the long term (months, years) or can contribute to delaying the aging process is not evident from the data. Significant changes in lipid metabolism in response to physical activity/exercise were also detected in the review article by Kelly et al., further metabolic changes are characterized by alterations in tricarboxylic acid cycle (TCA), amino acid metabolism and insulin sensitivity among others [133]. These changes are consistent and reflect the known physiological changes found in the literature during physical activity and/or exercise. The question arises which type of physical activity/exercise achieves the greatest health promoting effects and thus contributes to an improvement/maintenance of health status and thus possibly contributes to a prolongation of health status. The greatest metabolic effects are achieved by high intensity exercise. For this testing protocols two isocaloric training units of different intensities are often compared, e.g., an investigation on 10 well-trained triathletes during a high intensity interval training (HIIT) vs. moderate exercise session [134]. The testing was performed on two different days, with a minimum of 7 days between the two trials. The average intensity of the HIIT was 81.6 ± 3.7% VO_2max_, that of the moderate session 66.7 ± 3.5% VO_2max_. This showed particular differences in the current metabolic processes (aerobic vs. anaerobic energy supply), while other metabolic processes, such as serum free fatty acid concentrations, did not differ between the two interventions. They quantified a total of 49 metabolites, of which 11 showed changes after both exercise tests, 13 only after the HIIT session and 5 only after the moderate training session [134]. Such intensity-dependent changes are also found in other studies [130,135]. However, all the results considered in the previous section refer only to the current metabolic changes during and after sports. The actual positive health outcomes, which in most cases only manifest themselves after weeks, months or even years of regular physical activity with sufficient intensity, cannot be mapped.

There are far fewer studies in the literature found that attempt to detect these effects, sometimes also providing conflicting results. While the majority of studies clearly demonstrated altered metabolic state, a few studies showed no differences [133]. For example, the metabolite profile of a total of 216 overweight individuals was studied [136]. They were divided into different exercise groups and a control group. Metabolites were compared after a 24-week intervention period. This comparison did not show significant differences between the exercise and control groups; however, when a pre-post comparison was performed within the exercise group, changes in lipid metabolism and in cardiometabolic risk markers were detected. The results obtained were only adjusted for age, sex and baseline risk factors (e.g., body mass index (BMI) and fasting glucose) [136]. However, diet, which can also produce large short-term fluctuations in the metabolite profile, or other environmental influences were not taken into account, making the interpretation of the results difficult and leading to insufficient metabolic differences in the exercise vs. control group. Furthermore the comparison of 192 adolescents aged between 12 and 15 years could prove differences in the profile between males and females, but no differences in the metabolite profile between the physically active and inactive group [137]. The cross-sectional design of this study, which provided only a single sampling, seems to be a limitation. Moreover, daily fluctuations in the metabolite profile according to nutritional state were not taken into account, which could explain the inconclusive results [138].

These two examples show how study designs can affect the outcome of the study and highlight the need for standardization, not only of the analytical methods, but also of the interventions [139,140,141]. The fact that this is often associated with high financial, personnel and time costs, especially in long-term exercise intervention studies, makes them difficult to implement. The challenge of a large number of investigations is therefore that many parameters influencing the metabolome (e.g., environmental, nutritional, sex, age, drugs) are not taken into account in the evaluation of the results obtained and thus high quality results can often not be assessed [142]. A cross-sectional study design is also often chosen that use different criteria to classify subjects into categories of different performance [143,144]. This is achieved, for example, through the use of questionnaires on physical activity or the determination of VO_2max_. Even though data were statistically adjusted for age and gender the lack of an appropriate intervention is limiting these analysis.

In addition to the results presented, there are numerous other articles found in the literature that deal with metabolic changes in exercise [145,146,147] which shows the great potential of the method in the study of health and diseases and the impact of exercise intervention on metabolic pathways. However, the effects on the processes of cell proliferation, changes in gene activity can only be inadequately mapped. Hence, multiomics approaches will be necessary to fully elucidate the mechanisms triggered by exercise interventions.

## 5. Discussion

**Figure 3:**
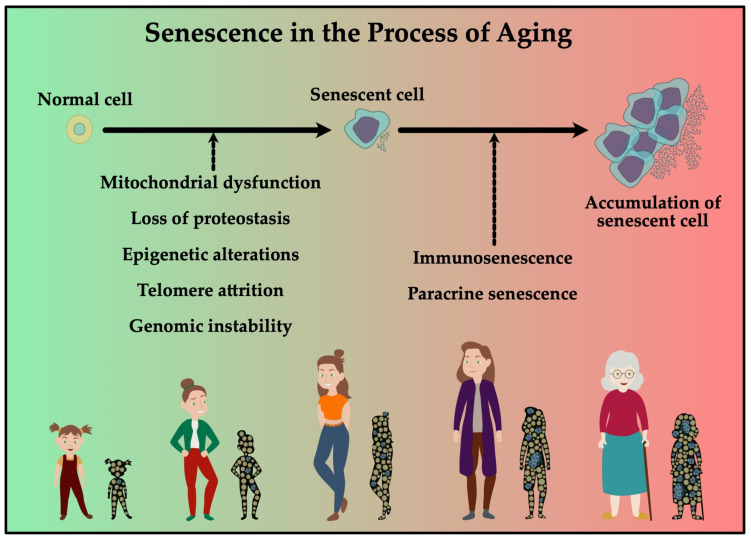
Normal cells can be induced to become senescent, which can induce paracrine senescence. Together with a decline in immune function, this could induce accumulation of senescent cells. In the elderly this accumulation contributes to increased risk in developing age-related diseases.

Figure 3 shows factors affecting senescence during aging such as telomere attrition, epigenetic alterations and genomic variability. The objective of research is to inhibit these processes to prolong the healthy lifespan. The underlying mechanisms are not yet fully understood and new analytical approaches are being developed to obtain a more comprehensive insight.

As shown in the previous chapters, the three analytical methods presented can be used to investigate different aspects of metabolic, genetic and epigenetic changes in human tissues. While the telomere length of leukocytes demonstrates correlations with physical fitness and activity in the elderly, it is currently too early to make general statements about a potential impact of specific exercise interventions on telomere length. This is particularly due to the difficulty of standardizing the study design and the frequently chosen cross-sectional studies. Another factor complicating the analysis and interpretation of the data is the interindividual variation in test results, making subject-specific predictions difficult. Direct correlations between the TL and the DNA methylation level [148,149] as well as the metabolite profile [150,151,152] exist and thus offer a new possibility to gain a deeper insight into the mechanisms of telomere shortening and its prevention. Although telomeric DNA-repeats themselves cannot be methylated, the degree of methylation of subtelomeric DNA plays a major role in TL. Hypermethylation of these regions results in shortening of telomeres and thus leads to premature aging of the cell. From these studies it can be concluded that interventions, which are able to influence the degree of methylation of the subtelomeric DNA, could also result in an elongation of the telomeres and have a protective effect against premature aging processes. Therefore, slowing of age-related methylation of subtelomeric DNA was already shown in tai chi practice but needs to be studied for other types of exercise [79].

The influence of exercise on TL, DNA methylation and prevention of DNA damage is demonstrated by the review of Sellami et al. which shows that particularly aerobic/endurance exercise has the potential in conserving TL and preventing DNA damage [91]. However, for anaerobic exercise, such as sprint training, the research data is not yet clear.

In addition to the combination of analysis of the two, TL and DNA methylation, the measurement of metabolic profiles can also provide a deeper understanding of the underlying mechanisms. A challenge of integrating those analytical approaches is the large amount of data obtained. Metabolomics for itself generate huge datasets displaying thousands of different metabolites per measurement, which can be identified and quantified only with advanced statistical analysis methods. Already the amount of data generated by the omics technologies is large enough that an evaluation and interpretation of the results is only possible with adequate software and bioinformatics solutions. A combination of two or more methods would make it necessary to develop statistical approaches combining them, or to modify existing ones for appropriate data evaluation. This has already been successfully implemented for various applications but requires further adaptation—especially when combining data from metabolomics, DNA methylation and telomere length determination.

## 6. Conclusions

As shown in Figure 1, exercise and/or regular physical activity as well as other lifestyle factors such as nutrition or sleep time and/or quality are simple ways to be implemented in daily life for retardation of the normal aging process, therefore extending lifespan. However, to what extent these interventions influence aging and the processes leading to accumulation of senescent cells in the elderly is still not fully understood. Therefore, the synergistic effects of the aforementioned methods might help to identify mechanisms of age-related damage. This opens a large field of possible applications in the prevention and treatment of pathological processes and could thus be a suitable method for implementing exercise and/or appropriate nutritional strategies for a longer and healthier life.
